# Comprehensive metabolomics study on the pathogenesis of anaplastic astrocytoma via UPLC-Q/TOF-MS

**DOI:** 10.1097/MD.0000000000029594

**Published:** 2022-08-05

**Authors:** Chao Du, Zhehao Huang, Bo Wei, Miao Li

**Affiliations:** Department of Neurosurgery, China-Japan Union Hospital of Jilin University, Changchun, Jilin.

**Keywords:** anaplastic astrocytoma, metabolomics, metabolism, biomarker, immune regulation

## Abstract

Anaplastic astrocytoma (AA) is a malignant carcinoma whose pathogenesis remains to be fully elucidated. System biology techniques have been widely used to clarify the mechanism of diseases from a systematic perspective.

The present study aimed to explore the pathogenesis and novel potential biomarkers for the diagnosis of AA according to metabolic differences. Patients with AA (n = 12) and healthy controls (n = 15) were recruited. Serum was assayed with untargeted ultraperformance liquid chromatography-quadrupole/time-of-flight-mass spectrometry (UPLC-Q/TOF-MS) metabolomic techniques. The data were further evaluated using multivariate analysis and bioinformatic methods based on the KEGG database to determine the distinct metabolites and perturbed pathways.

Principal component analysis and orthogonal projections to latent structures-discriminant analysis (OPLS-DA) identified the significance of the distinct metabolic pattern between patients with AA and healthy controls (*P* < .001) in both ESI modes. Permutation testing confirmed the validity of the OPLS-DA model (permutation = 200, Q2 < 0.5). In total, 24 differentiated metabolites and 5 metabolic pathways, including sphingolipid, glycerophospholipid, caffeine, linoleic acid, and porphyrin metabolism, were identified based on the OPLS-DA model. 3-Methylxanthine, sphinganine, LysoPC(18:1), and lactosylceramide were recognized as potential biomarkers with excellent sensitivity and specificity (area under the curve > 98%).

These findings indicate that the perturbed metabolic pattern related to immune regulation and cellular signal transduction is associated with the pathogenesis of AA. 3-Methylxanthine, sphinganine, LysoPC(18:1), and lactosylceramide could be used as biomarkers of AA in future clinical practice. This study provides a therapeutic basis for further studies on the mechanism and precise clinical diagnosis of AA.

## 1. Introduction

Anaplastic astrocytoma (AA) is a type of diffusely infiltrating malignant astrocytoma.^[1]^ Nuclear atypia, increased cellularity, and absence of the pathologic hallmarks of glioblastoma are the characteristic features of AA.^[2]^ The pathogenesis of AA is markedly associated with molecular immunological mechanisms such as macrophages and T cell activation,^[3,4]^ and biochemical reactions in vivo.^[5]^ Regulation of AA has been confirmed to be related to various inflammatory and immunological pathways.^[6,7]^ Moreover, the mechanism has been demonstrated to be closely associated with nutrient metabolism and epigenetic regulation.^[8,9]^ Although mutations in the IDH gene have been confirmed as one of the critical factors of AA,^[10]^ a limited understanding of its mechanism is not sufficient to provide more support for precise diagnosis and treatment in clinical practice.

System biology methods have been widely used to explore the pathogenesis of various diseases in recent years.^[11,12]^ Metabolomics, as a novel omics technology, focuses on small-weight molecules <1 kDa, and is considered the best indicator of the inner mechanism of diseases.^[11]^ High-resolution mass spectrometry-based untargeted metabolomics is emergingly used to reveal novel biological significance.^[13]^ Recently, several metabolomics studies have been performed to illustrate the pathogenesis of glioma^[14]^; however, the majority of these studies only examined the metabolic pattern of glioblastoma or the profile in vitro.^[15]^ The most recent study has also only focused on the metabolic differences between low- and high-grade glioma specimens.^[16]^ A noninvasive identification of prediagnostic metabolic patterns for AA is urgently required to improve the therapeutic strategy of AA. Systematic metabolomics studies on the pathogenesis of AA are rarely reported.

Therefore, the present study recruited patients with AA and explored their metabolic profiles based on ultraperformance liquid chromatography-quadrupole/time-of-flight-mass spectrometry (UPLC-Q/TOF-MS). The hypothesis that there were significant perturbations in the metabolic profile of patients with AA compared with that of healthy controls (HCs) was evaluated. In addition, the differentiated metabolites have the potential to be biomarkers for the diagnosis of AA.

## 2. Materials and Methods

### 2.1. Subjects recruitment and ethics assessment

Patients with AA were recruited from the China-Japan Union Hospital of Jilin University. The patients were diagnosed with computed tomography and nuclear magnetic resonance imaging as AA for the first time, and finally confirmed with pathological sections of the tumor tissue after surgical operations. The age of the patients ranged from 50 to 60 years, with the gender balanced. Age- and sex-matched volunteers of the HC group were also recruited. The HC subjects were not diagnosed with any diseases and did not have any history of neurological diseases. Current smokers or those with a history of smoking were excluded. All subjects provided written informed consent. This study was approved by the China-Japan Union Hospital Ethics Committee of Jilin University (approval number: 2018-NSFC-003) and was registered at the International Clinical Trials Registry Platform and Chinese Clinical Trial Registry (no. ChiCTR1900024766).

### 2.2. Patient sample collection and preparation

Peripheral venous blood was obtained from all subjects after a 12-h fasting period when they were first diagnosed. A total of 10 mL blood was collected into coagulation-promoting tubes for serum separation and then stored at –80°C for further processing. The serum samples were thawed on ice and mixed with methanol (1:3, v/v; cat. no. A4521; Thermo Fisher Scientific, Inc.). The mixture was vortexed for 3 minutes, incubated on ice for 15 minutes, and centrifuged at 4°C and 10,000 × *g* to remove the protein content from the samples. The supernatant (without protein) of all samples was lyophilized (12N-60A model, HNZXIB, Inc.) at –60°C and 10 Pa air pressure for ≥24 hours. The residues were redissolved in 100 μL of methanol-water (4:1, v/v). A quality control (QC) sample was prepared by pooling 20 μL from each sample.

### 2.3. UPLC-Q/TOF-MS assay

All samples were injected into the UPLC system (ACQUITY; BEH C18 column, 2.1 mm × 100 mm, 1.7 mm; Waters Corporation) and Q/TOF-MS (Xevo G2-S; Waters Corporation). The conditions and parameters of the chromatographic systems were optimized as described previously.^[17]^ In brief, the temperatures of the BEH column and autosampler were 30°C and 15°C, respectively. Mobile phase A (0.1% formic acid; cat. no. PI85170; Thermo Fisher Scientific, Inc.) and B (0.1% formic acid in acetonitrile; cat. no. A998-4; Thermo Fisher Scientific, Inc.) was used to elute from 90% to 10% (mobile phase A) at a flow rate of 0.4 mL/min. The collection modes of the spectrometry included positive electrospray ionization (ESI^+^) and negative electrospray ionization (ESI–). Consecutive injections of aliquots of the QC sample were performed to ensure the stability and precision of the system before injecting the analytes. In addition, another 6 aliquots of the QC sample were inserted randomly throughout the sample lists of the 2 ESI modes.

### 2.4. Data processing

The alignment, deconvolution, and reduction of the raw MS data were first performed with MassLynx software (v4.1; Waters Corporation) to align the retention time and mass peaks of all samples for further analysis. The main processing parameters were set as follows: The involved retention time of the data was from 0 to 29 min. All metabolic features were captured from 100 Da to 1300 Da, with a mass tolerance of 0.10 and a minimum peak intensity of 0.5 minutes. Noise was eliminated at level 6. Other parameters were described in a previous report (9). The data were analyzed primarily with MarkerLynx (v4.1; Waters Corporation) and then exported to SIMCA P (v14.1; Umetrics) for further multivariate analysis. Principal component analysis (PCA) and orthogonal projections to latent structures-discriminant analysis (OPLS-DA) models were established. The metabolic features associated with the distinct metabolic profiles were summarized to identify the metabolites.

### 2.5. Compound identification

The remarkably distinct metabolic features (variable importance of project > 1 and *P* < .05 between the groups) were identified based on their precise molecular mass weight according to the Human Metabolites Database (HMDB, v4.0).^[18]^ Then, the identified metabolites were further firmly determined by matching the tandem MS/MS fragments with the HMDB and METLIN databases.^[19]^ Metabolites with a mass molecular weight error of < 15 ppm were included. Adducts of the metabolites were M + H⌉^+^ and M + Na⌉^+^ for ESI^+^, and M-H⌉^−^ and M + FA⌉^−^ for ESI ^−^ according to the chemical composition of the solvent and the mobile phases.

### 2.6. Bioinformatic analysis

The perturbed metabolic pathways were identified based on the MetaboAnalyst 4.0 platform (https://www.metaboanalyst.ca/).^[13]^ Related metabolic functions were enriched via the Network Explorer module. The sensitivity and specificity of the potential metabolic markers from the OPLS-DA models were examined by receiver operating curve (ROC) with the pROC package (https://cran.r-project.org/web/packages/pROC/index.html). Evaluation of the stability and suitability of the UPLC-Q/TOF-MS system relied on the relative standard deviation of the QC samples. Interaction among the distinct metabolites was identified based on the Kyoto Encyclopedia of Genes and Genomes (updated on Jun 25, 2019) database,^[20]^ while the interaction network was established using Cytoscape software (v3.7.1).^[21]^

### 2.7. Statistical analysis

Statistical analysis of the homogeneity of variance and normality of the data was performed first with F and Kolmogorov-Smirnov tests. The data set that did not meet the normality criteria was analyzed with the Mann-Whitney-Wilcoxon test for statistical significance. Student *t* test and Welch *t* test were used to analyze the data with or without homogeneity of variance, respectively. *P* < .05 was considered to indicate a statistically significant difference. Statistical and bioinformatic analyses were conducted using R software (v3.6.1).

## 3. Results

Univariate and multivariate statistical analyses were performed. Significant differences in metabolic profile, metabolite biomarkers, and metabolic pathways were reported.

### 3.1. Clinical characteristics of all participants

In total, 12 patients with AA and 15 age- and sex-matched HC subjects were included in the present study. The clinical demographics of the participants are summarized in Table 1. The living status, as indicated by the Karnofsky Performance Scale index showed a relatively normal living status of patients with AA, although it was remarkably lower than that of the HC group (*P* < .05). In addition, the tumor location of AA and the IDH phenotypes among the AA samples were distributed evenly and did not display a significant bias. The cumulative dosage of steroid used in the previous 3 months was equivalent to < 2000 μg beclomethasone propionate.

**Table 1:** Characteristics of the subjects

Groups/indexes	Anaplastic astrocytoma	Healthy control	*P*
Number	12	15	>.05
Gender (F/M)	6/6	7/8	>.05
Age	58.42 ± 4.48	60.02 ± 5.31	>.05
BMI	22.33 ± 2.87	21.80 ± 3.51	>.05
KPS	76.25 ± 11.01	98.06 ± 2.25	<.01
Courses/Months	3.73 ± 1.71	NA	NA
Grade	III	NA	NA
Tumor Localization (P/T/F)	4/4/4	NA	NA
*IDH-WT/MT/NOS*	4/5/3	NA	NA
Steroid Usage	4	2	>.05

### 3.2. Evaluation of the quality of the UPLC-Q/TOF-MS system

**Figure 1.**
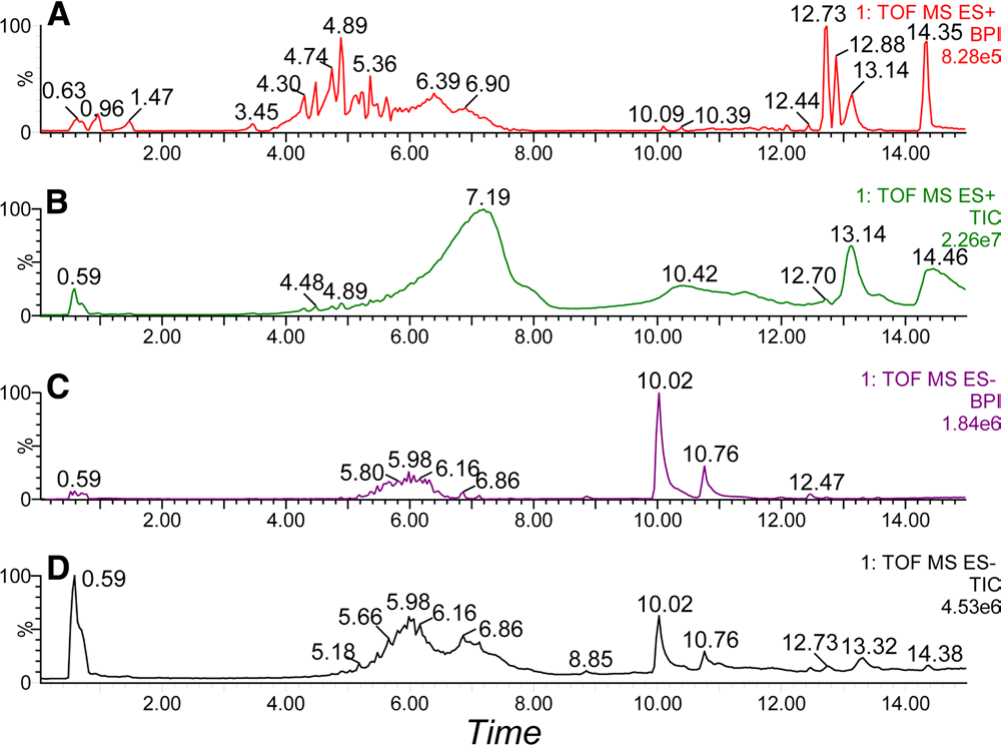
Chromatographic plots of the serum extraction sample under different ESI modes (ESI ^+^ and ESI–). (A) Base peak ionization and (B) total ionization chromatography plots of serum under ESI ^+^ mode. (C) Base peak ionization and (D) total ionization chromatography plots of serum under ESI– mode. The metabolites peaks were eluted and separated clearly and evenly. ESI, electrospray ionization.

The chromatographic peak plots, including the total ion chromatogram and base peak intensity in ESI ^+^ and ESI– modes are displayed in Figure 1. The molecules were eluted successively. Evaluation of the stability and suitability of the UPLC-Q/TOF-MS system was first performed based on the QC sample. A total of 10 abundant ion features were selected randomly from the dataset table from 200 to 1000 kDa in both modes. The retention times, peak areas, and masses of the 20 ions were assessed as critical indices. The relative standard deviation of repeatability and the intermediate precision of the retention time, peak area, and mass were < 1.0% and displayed remarkable stability, particularly the retention time and mass (<0.001%) (Table 2).

**Table 2:** Evaluation on the stability of the chromatographic and spectrometry (UPLC-Q/TOF-MS) system

ESI Modes	Ion features (RT_MASS)	RT (RSD/%)		Peak area (RSD/%)		Mass (RSD/%)	
		Rep	Pre	Rep	Pre	Rep	Pre
+	0.54_300.0396	0.00320	0.00100	0.06	0.16	0.000012	0.000065
	11.48_328.2453	0.00021	0.00310	0.12	0.35	0.000030	0.000032
	0.80_418.1915	0.00005	0.00290	0.06	0.17	0.000010	0.000004
	6.54_508.9713	0.00068	0.00010	0.09	0.68	0.000004	0.000027
	20.55_551.3241	0.00031	0.00050	0.63	0.45	0.000040	0.000031
	26.25_672.4604	0.00047	0.00600	0.41	0.33	0.000001	0.000007
	6.87_724.7766	0.00072	0.00040	0.02	0.54	0.000001	0.000007
	7.04_820.7493	0.00168	0.00120	0.03	0.76	0.000004	0.000002
	14.57_966.3624	0.00096	0.00680	0.10	0.49	0.000031	0.000048
	6.90_1105.6546	0.00071	0.00037	0.74	0.36	0.000002	0.000003
-	9.95_220.0499	0.00063	0.00023	0.28	0.06	0.000003	0.000001
	21.56_391.4096	0.00214	0.00281	0.63	0.08	0.000001	0.000016
	16.80_429.4010	0.00364	0.00098	0.49	0.01	0.000001	0.000000
	25.39_511.4481	0.00210	0.00038	0.10	0.54	0.000002	0.000011
	25.53_567.4573	0.00057	0.00003	0.26	0.31	0.000021	0.000023
	20.56_610.3386	0.00127	0.00091	0.37	0.13	0.000035	0.000004
	5.98_672.1523	0.00001	0.00010	0.56	0.25	0.000048	0.000002
	5.60_757.6745	0.00007	0.00035	0.64	0.29	0.000029	0.000001
	25.17_824.3254	0.00089	0.00064	0.51	0.05	0.000034	0.000012
	11.93_922.2472	0.00037	0.00015	0.44	0.18	0.000038	0.000000
Maximun RSD/%	NA	**0.00364**	**0.00680**	**0.74**	**0.76**	**0.000048**	**0.000065**

**Figure 2.**
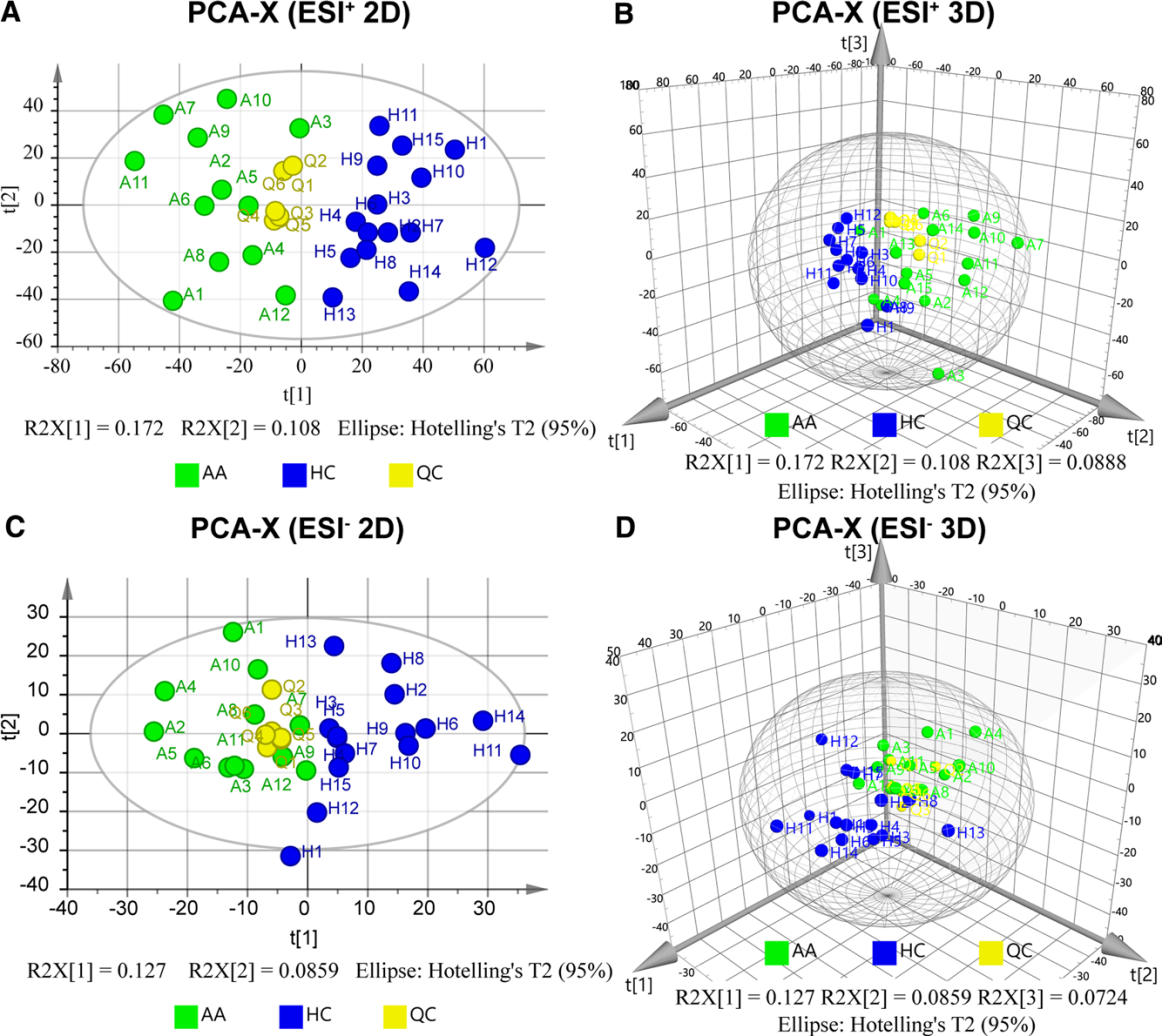
PCA result of ESI ^+^ and ESI–. (A) PCA plot of ESI ^+^ in (2D). (B) PCA plot of ESI ^+^ in 3 dimensions (3D). (C) PCA plot of ESI– in 2D. (D) PCA plot of ESI– in 3D. (PC1 = 0.172, PC2 = 0.108, PC3 = 0.0889 for ESI^+^; PC1 = 0.127, PC2 = 0.0859, PC3 = 0.0724 for ESI–). AA was further abbreviated as A, HC as H and QC as Q in the plots above. The metabolic patterns of the different groups in both ESI ^+^ and ESI– modes were significantly distinct. QC samples were clustered at the center of the plots. PCA, principal component analysis; ESI, electrospray ionization; 2D, 2 dimensions; 3D, 3 dimensions; QC, quality control; HC, healthy control; AA, anaplastic astrocytoma.

PCA, an unsupervised clustering model, displayed the relative similarity of the samples (Fig. 2). Nearly all the samples were included in the ellipse of the plot, which indicated the absence of a significant abnormal sample (the confidence interval was 95%). The PCA of ESI ^+^ consisted of 5 principal components with a sum of 68.24%, while the PCA of ESI– was formed by 6 main components and summed 64.68%. The PCA plots represented the major features of the metabolic patterns of all the samples. There was a significant separation between the AA and HC groups. Moreover, the QC sample aliquots distributed throughout the entire injection list were tightly clustered at the center of the plot, and displayed adequate stability and repeatability. In addition, a batch effect did not exist, according to the clustered QC sample points.

**Figure 3.**
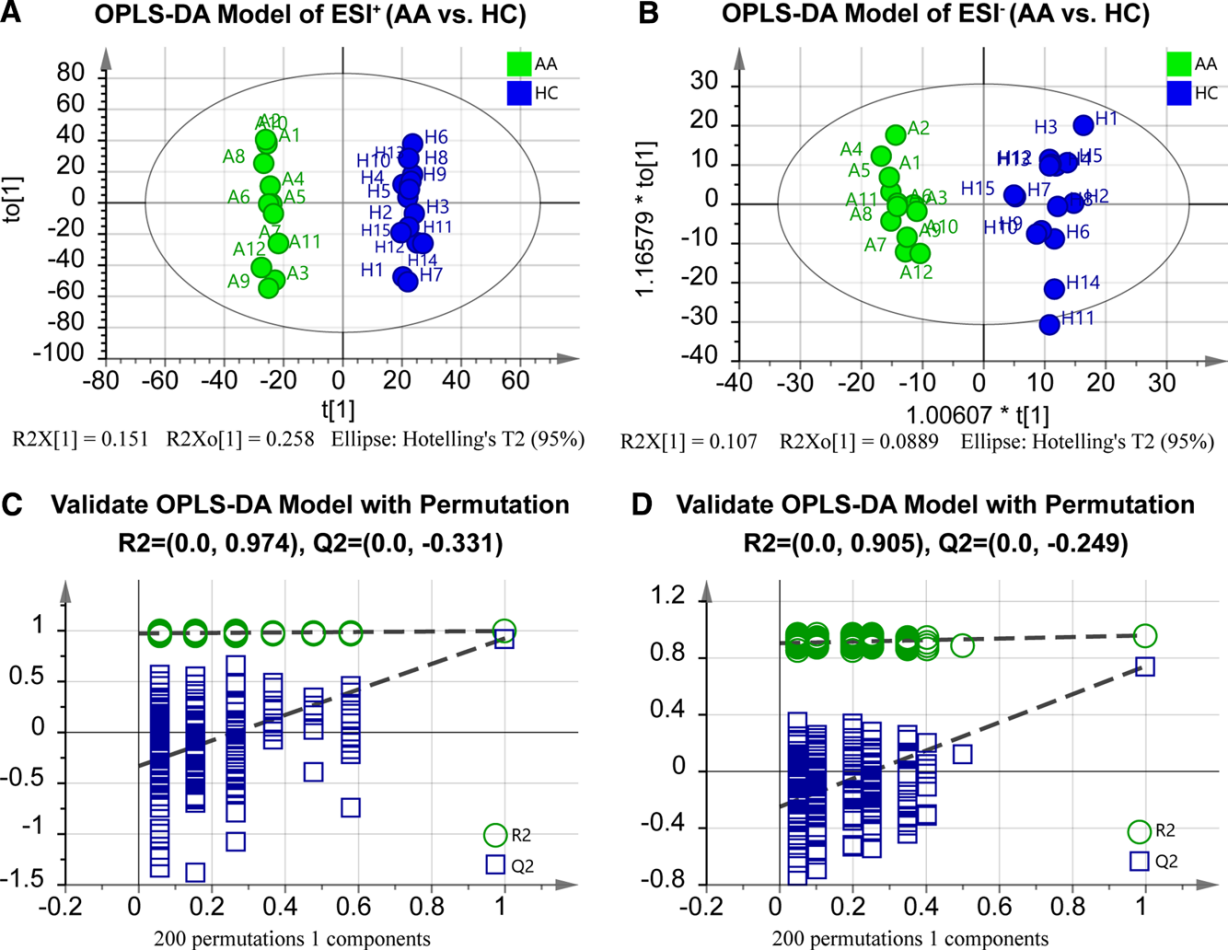
OPLS-DA models and validation plots. (A) OPLS-DA model for AA vs HC in ESI ^+^ mode (*P* < .0001, Coefficient of Variation, CV-ANOVA) and (B) its validation with permutation (R2 = 0.974, Q2 = -0.331). (C) OPLS-DA model for AA vs HC in ESI– mode (*P* < .001, CV-ANOVA) and (D) its validation with permutation (R2 = 0.905, Q2 = -0.249). The discriminant models displayed significant separation between AA and HC. Permutation test showed a high confidence about the validity of the OPLS-DA models. OPLS-DA, orthogonal projections to latent structures-discriminant analysis; ESI, electrospray ionization; HC, healthy control; AA, anaplastic astrocytoma.

OPLS-DA models based on PCA were further built to discriminate significant differences between the AA and HC groups. A total of 4386 metabolic features in ESI ^+^ and 1942 in ESI– were included in the OPLS-DA models. As shown in Figure 3A and B, the samples of the groups were significantly located on different sides of the plot, and displayed notably explicit separation, indicating that remarkably different metabolic features existed. To avoid overfitting errors, the OPLS-DA models were validated with permutation. As a result, the grouping sample lines were located underneath the random sampling lines (Fig. 3C and D). The Q2 values of the permutation tests were < 0.05. Therefore, these OPLS-DA models were evidently reliable for the identification of characteristic metabolite biomarkers (*P* < .001).

### 3.3. Identification of distinct metabolites and their levels

**Figure 4.**
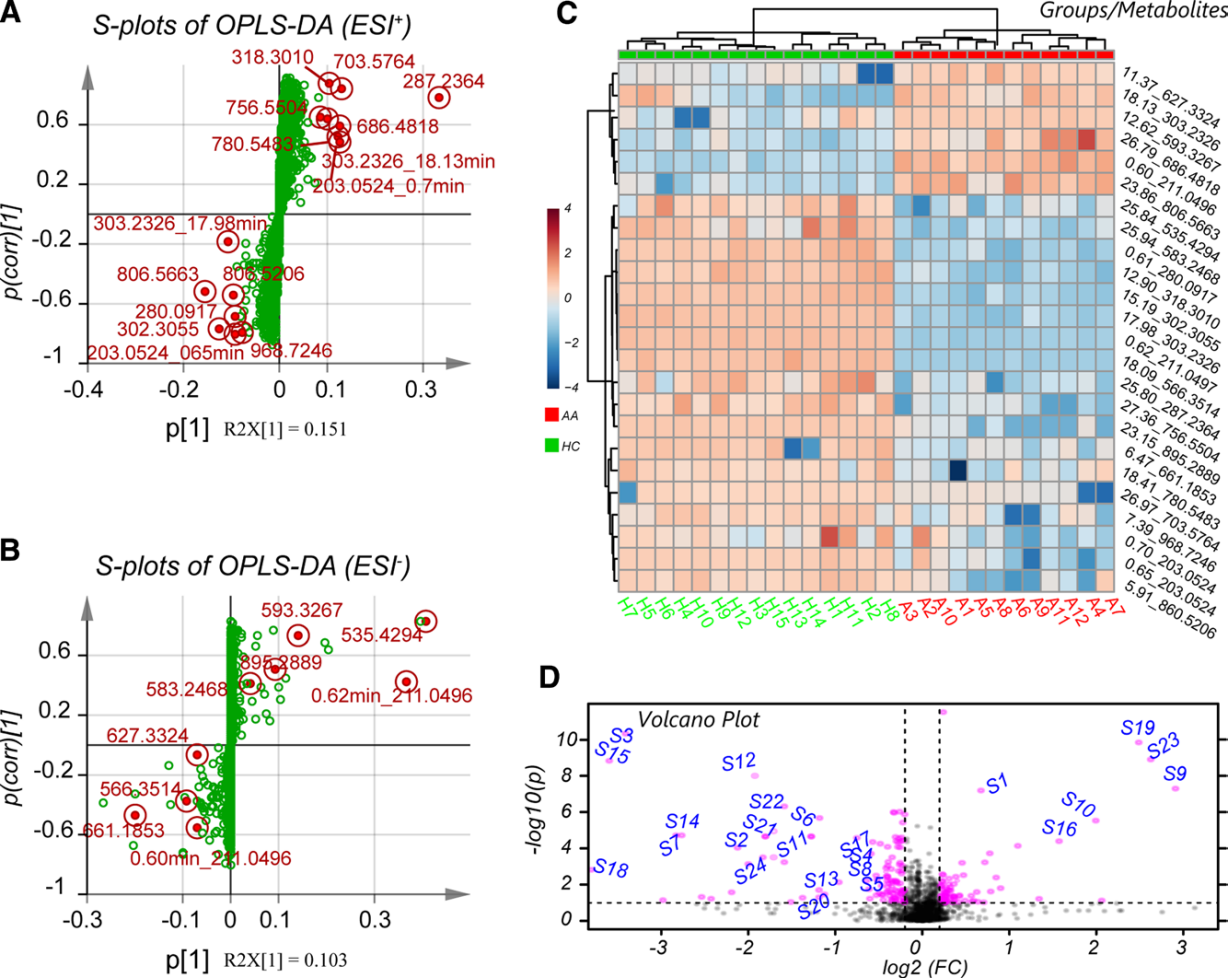
Identified multivariate and content level of the differentiated metabolites. S-plots of the orthogonal projections to latent structures discriminant analysis model in (A) ESI ^+^ and (B) ESI– modes. All distinct metabolites were highlighted as red circles. The other metabolic features were displayed as green points. Metabolites of both ESI modes were located on the edge of the S-plots, which indicated a high correlation and covariance. (C) Heatmap displayed the relative content level of all perturbed metabolites in both ESI modes. The samples of both groups were naturally clustered into their biological groups, indicating an exemption of the abnormal samples and excellent representativeness of metabolic distinction between the groups. (D) Volcano plot of the FC and P-values of all metabolites after logarithmic transformation. All the differentiated metabolites features identified scattered largely with significant FC and/or P-values and were marked with their labels. ESI, electrospray ionization; FC, fold change.

In total, 24 metabolites were identified based on the OPLS-DA model. Detailed information about the identified metabolites is summarized in Table 3. The metabolites were distributed in S-plots (Fig. 4A and B). All of them were located on the edge of the plot, which indicated potentially high correlation and covariance (Table 4). The relative content level and fold change of these metabolites are displayed as a heatmap and a volcano plot in Fig. 4C and D, respectively. All samples were clustered into their own biological groups, which indicated their potential to be used as biomarkers.

**Table 3:** Metabolites identified in serum based on the discriminant models

No.	Compounds	Retention Time/min	Mass/Da	Formula	Characteristic fragments	ESI mode	Error/ppm	Metabolic pathways
S1	Xanthine	0.60	211.0496	C5H4N4O2	41.998, 65.0140, 108.0198, 133.0150, 151.0258	–	1	Caffeine
S2	Glycerophosphocholine	0.61	280.0917	C8H20NO6P	57.0335, 86.0964, 136.9998, 240.0995, 258.1101	+	1	Glycerophospholipid
**S3**	3-Methylxanthine	0.62	211.0496	C6H6N4O2	41.9985, 93.0020, 122.0354, 165.0413	–	1	Caffeine
S4	Theobromine	0.65	203.0524	C7H8N4O2	83.0609, 96.0086, 138.0304, 181.0726	+	8	Caffeine
S5	Paraxanthine	0.70	203.0524	C7H8N4O2	67.0313, 69.0466, 96.0575, 124,0522, 181.0725	+	8	Caffeine
S6	PS(18:0/22:5)	5.91	860.5206	C46H80NO10P	267.0980, 317.4320, 664.3065, 728.1304, 859.5243	+	14	Glycerophospholipid
S7	Heme	6.47	661.1853	C34H32FeN4O4	41.0236, 59.0234, 527.1903, 597.1595	–	5	Porphyrin
S8	Galabiosylceramide (d18:1/22:0)	7.39	968.7246	C52H99NO13	88.0975, 345.9200, 471.0752, 810.4105, 903.0735	+	4	Sphingolipid
S9	Protoporphyrinogen IX	11.37	627.3324	C34H40N4O4	44.9977, 463.2862, 505.2986, 549.2890, 567.2971	–	2	Porphyrin
S10	L-Urobilin	12.62	593.3267	C33H46N4O6	27.6384, 185.3496, 447.1560, 594.3721	–	3	Porphyrin
S11	Phytosphingosine	12.9	318.301	C18H39NO3	62.0606, 113.1330, 197.2269, 268.2640, 318.3008	+	2	Sphingolipid
**S12**	Sphinganine	15.19	302.3055	C18H39NO2	127.1487, 141.1643, 155.1800, 169.1956, 284.2953	+	0	Sphingolipid
S13	PC(16:0/16:0)	17.36	756.5504	C40H80NO8P	184.0739, 478.3297, 551.5039, 675.4965, 734.5700	+	1	Linoleic acid
S14	Bovinic acid	17.98	303.2326	C18H32O2	121.1012, 191.1794, 221.2264, 235.2420, 263.2369	+	10	Linoleic acid
**S15**	LysoPC(18:1)	18.09	566.3514	C26H52NO7P	59.0136, 78.9599, 122.9853, 173.0228	–	9	Glycerophospholipid
S16	Linoleic acid	18.13	303.2326	C18H32O2	111.1168, 137.1325, 151.1481, 235.2420, 281.2475	+	10	Linoleic acid
S17	Sulfogalactosylceramide	18.41	780.5483	C40H77NO11S	43.0651, 82.7094, 279,0456, 642.0731, 779.4318	+	5	Sphingolipid
S18	Uroporphyrinogen III	23.15	895.2889	C40H44N4O16	59.0133, 626.1965, 747.2877, 773.2408	–	0	Porphyrin
**S19**	Lactosylceramide (d18:1/12:0)	23.86	806.5663	C42H79NO13	163.0601, 282.2791, 446.4356, 606.3848, 770.5413	+	5	Sphingolipid
S20	Vitamin A	25.8	287.2362	C20H31O	69.0704, 107.0861, 133.1017, 189.1643, 269.2269	+	3	Retinol
S21	Beta-carotene	25.84	535.4294	C40H56	201.1643, 253.1597, 293.2283, 399.3671,519.4003	–	8	Retinol
S22	Bilirubin	25.94	583.2468	C33H36N4O6	134.0606, 241.1341, 285.1330, 446.2085, 565.2453	–	6	Porphyrin
S23	PE(18:3/14:0)	26.79	686.4818	C37H68NO8P	44.0494, 251.2733, 287.2369, 448.3186, 691.5061	+	9	Glycerophospholipid
S24	Sphingomyelin	26.97	703.5764	C39H80N2O6P	86.0231, 183.0475, 186.0742, 732.0098	+	1	Sphingolipid

**Table 4:** Change fold and multivariate statistics of the perturbed metabolites

Metabolites	Relative comparison	Fold change (AA/HC)	Log2(FC)	Covariance	Correlation
S1	AA > HC	1.5958	0.6743	–0.069700	–0.557837
S2	AA < HC	0.3207	–1.6406	–0.156779	–0.512242
S3	AA < HC	0.0888	–3.4940	0.039407	0.408745
S4	AA < HC	0.5932	–0.7534	–0.093510	–0.804158
S5	AA < HC	0.7430	–0.4286	0.123863	0.52474
S6	AA < HC	0.4831	–1.0495	–0.095251	–0.547364
S7	AA < HC	0.1463	–2.7729	–0.198145	–0.467091
S8	AA < HC	0.6415	–0.6406	–0.079232	–0.787085
S9	AA > HC	7.8625	2.9750	–0.069506	–0.0693994
S10	AA > HC	4.0314	2.0113	0.142604	0.727986
S11	AA < HC	0.4408	–1.1819	0.103230	0.871756
S12	AA < HC	0.2991	–1.7416	–0.125151	–0.764926
S13	AA < HC	0.5247	–0.9304	0.086365	0.655545
S14	AA < HC	0.1481	–2.7553	0.123863	0.52474
S15	AA < HC	0.0511	–3.5898	–0.091843	–0.373945
S16	AA > HC	2.6543	1.4083	0.123863	0.52474
S17	AA < HC	0.6174	–0.6958	0.100587	0.640483
S18	AA < HC	0.0293	–4.0932	0.365943	0.423538
S19	AA > HC	5.4326	2.4416	–0.156779	–0.512242
S20	AA < HC	0.4923	–1.0223	0.335006	0.784899
S21	AA < HC	0.3468	–1.5280	0.405920	0.82348
S22	AA < HC	0.3500	–1.5145	0.039407	0.408745
S23	AA > HC	7.0080	2.8090	0.125187	0.594305
S24	AA < HC	0.3058	–1.7092	0.128666	0.834996

### 3.4. Mining of biomarkers

**Figure 5.**
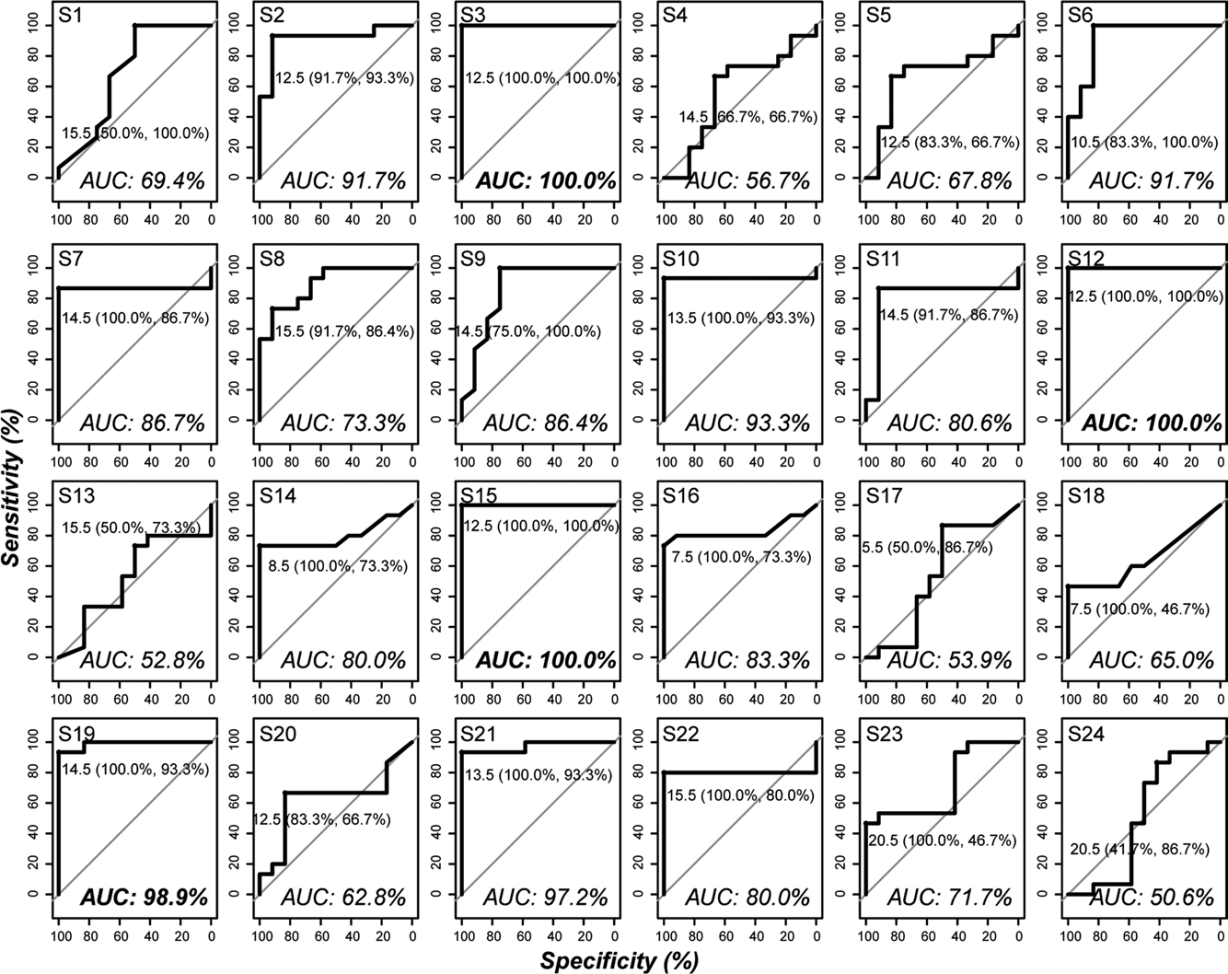
Receiver operating curve analysis results of all distinct metabolites for biomarkers mining. The metabolites S3, S12, S15 and S19 showed marked potential as biomarkers with extremely high precision (AUC > 98%), while some metabolites, including S2, S6, S10, and S21, have a relatively high potential to be used as biomarkers (98%>AUC > 90%). AUC, area under the curve.

The above 24 metabolites were evaluated for their potential as biomarkers with ROC. As shown in Figure 5, 8 metabolites passed the examination with a high sensitivity and specificity (area under the curve > 90%). Among them, 4 metabolites, (S3) 3-methylxanthine, (S12) sphinganine, (S15) LysoPC(18:1), and (S19) lactosylceramide (d18:1/12:0) could be used as reliable biomarkers because of their markedly high sensitivity and specificity (area under the curve > 98%).

### 3.5. Global profile of perturbed metabolic pathways and integrated network

**Figure 6.**
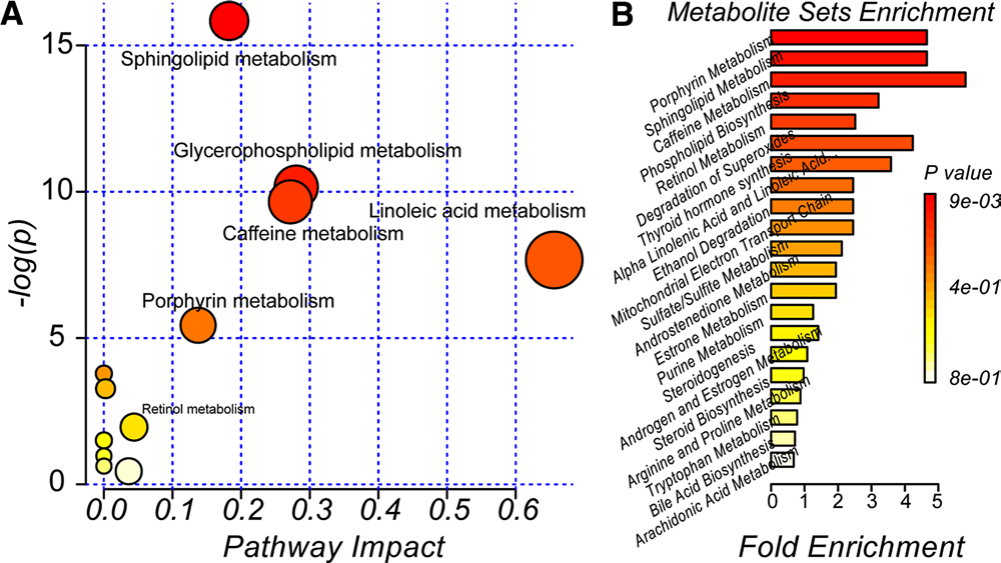
Metabolic pathways and functions enrichment from MetaboAnalyst 4.0 platform. (A) The statistical result of the impact extent of all the perturbed metabolic pathways. A total of 12 metabolic pathways were reported. However, only 5 pathways, including Sphingolipid, Glycerophospholipid, Linoleic acid, Caffeine and Porphyrin pathways (impact *>* 0.1, -log(P) > 5) were identified as perturbed significantly. (B) Metabolic functions enrichment based on the metabolites identified. Several top metabolic functions were also observed. DOS, degradation of superoxide; THS, thyroid hormone synthesis; ALALA, α-linolenic acid and linoleic acid; METC, mitochondrial electron transport chain.

The 24 metabolites identified above involved 6 metabolic pathways. Five of these, including sphingolipid, glycerophospholipid, linoleic acid, caffeine, and porphyrin metabolism, were perturbed significantly (impact > 0.1, –log(P) > 4.0). The extent of perturbation of these metabolic pathways is shown in Fig. 6A. Although retinol metabolism-related metabolites were identified in the present study, this pathway was not significantly perturbed (impact < 0.1, –log(P) < 4.0). The related pathways of all the metabolites involved are presented in Table 3. Besides, the metabolic functions of these metabolites were enriched (Fig. 6B), and the top enriched metabolic functions of these metabolites were nearly in complete agreement with the results of pathway prediction.

**Figure 7.**
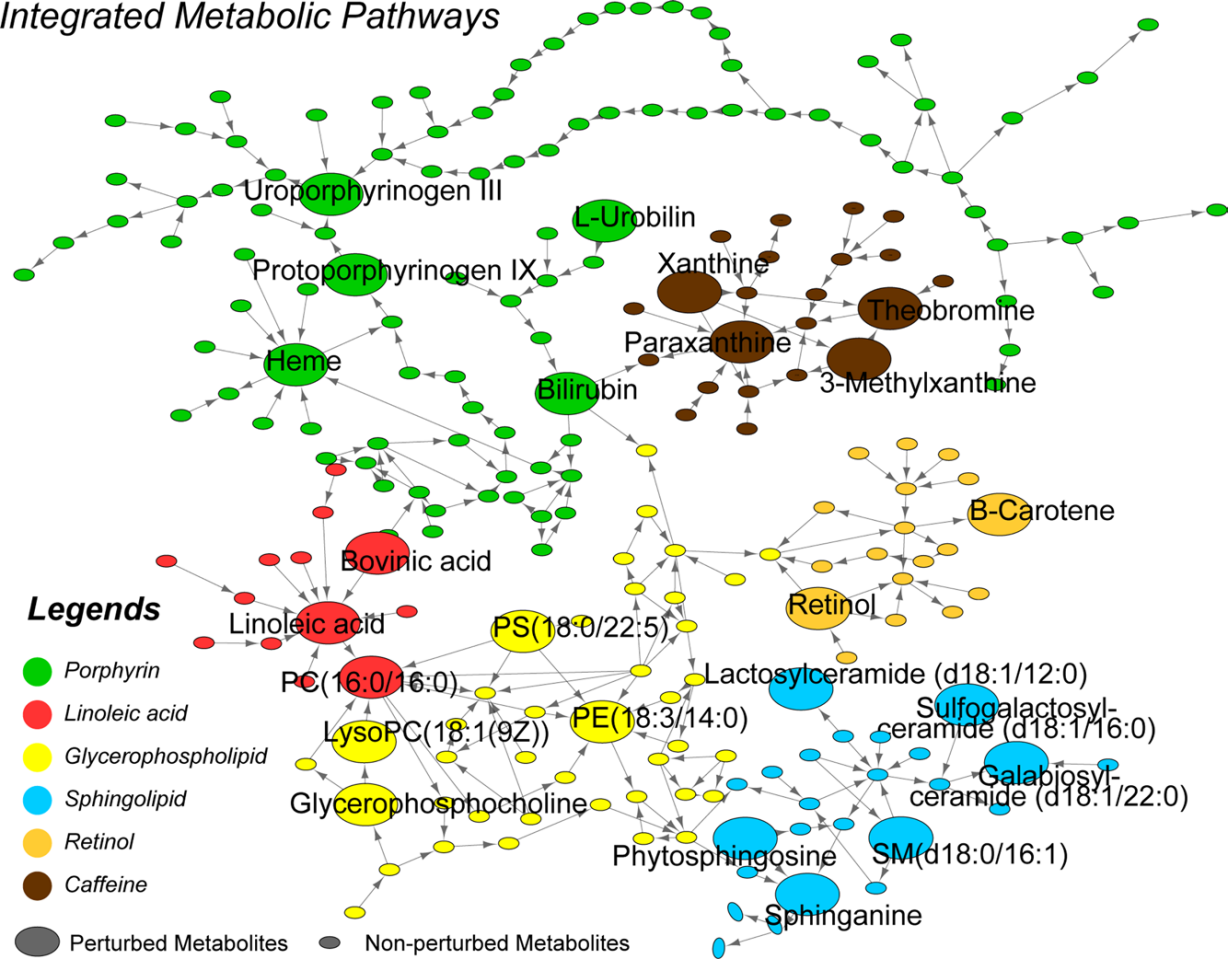
Interaction network of remarkably perturbed metabolic pathways and the distinct metabolites identified. Different metabolic pathways were clustered and shown in different colors, with the critical metabolites highlighted as large nodes. The various metabolic pathways formed an integrated network and generally described the systematic metabolic perturbation and the interaction of all critical metabolites.

Systematic metabolic interactions were integrated and constructed, as shown in Fig. 7. Different metabolic pathways were clustered and had various connections with other pathways. The network of all metabolites directly displayed the systematic perturbation and the related critical metabolites, and comprehensively revealed the changed metabolic profile of AA.

## 4. Discussion

Astrocytoma is a type of glioma that easily relapses after surgical operation with poor prognosis. The 2-year survival rate is <30%.^[22]^ Previous studies have reported that the pathogenesis of AA is associated with environmental factors as well as epigenetic and immune regulation.^[3,23,24]^ Recently, metabolomics techniques have been used to explore the mechanisms of various diseases due to their characteristics of the focused objects and metabolites. In the present study, UPLC-Q/TOF-MS was used to explore metabolic features. The differences in metabolic profiles between patients with AA and HC subjects were compared using multivariate analysis. A total of 24 distinct metabolites were identified, and 4 metabolites were confirmed to be biomarkers for diagnosis with markedly high precision. Furthermore, 5 significantly perturbed metabolic pathways (Sphingolipid, Glycerophospholipid, Linoleic acid, Caffeine, and Porphyrin metabolism) were reported. The detailed roles of these pathways in the pathogenesis of AA are discussed in detail below.

Metabolomics has been widely used to study the pathogenesis of various types of glioma, such as glioblastoma^[25]^ and glioma with different grades.^[16,26]^ Several prediagnostic markers have been associated with glioblastoma risk, including α- and γ-tocopherols.^[27]^ Differentiated choline and amino acids were found in glial tumor tissues of different grades.^[16]^ In addition, a multiomics study on glioblastoma in vitro revealed an association between genes and metabolic features.^[28]^ However, little is known about the underlying systematic metabolic alterations associated with the aggressive process of AA. Until now, more molecular pathogenetic evidence has clarified the promoting role of IDH mutation in the pathogenesis of glioma, while brain glioma containing mutated IDH could also give rise to specific metabolic signatures.^[29]^ In details, IDH mutation could cause competitive inhibition of α-ketoglutarate-dependent dioxygenase TET2 via oncometabolite-2′-hydroxgluterate.^[30]^ As a result, the whole genome of the tumor cell shows a global hypermethylation status, including O^6^-Methylguanine-DNA Methyltransferase (MGMT) promoter.^[31]^ The methylation of MGMT is highly associated with the effect of chemotherapy with temozolomide.^[32]^ Therefore, in the present study, the potential risk of difference caused by IDH mutation was balanced. Long-term metabolic reprogramming is a result of adaptive alterations of brain tumor cells, which involve changing lipids, amino acids, nucleic acids, and other metabolites necessary for cellular proliferation, tumor growth, and survival.^[15]^ In the present study, 5 significantly perturbed metabolic pathways were identified, with their roles explained as follows.

It has been reported that sphingolipids, glycerophospholipids, and linoleic acid could participate in the processes of energy metabolism and immune and inflammatory regulation,^[33,34]^ which indicates a potential association with the pathogenesis of AA.^[35]^ Albers et al reported increased levels of glycerophospholipid compounds with proton-decoupled [31]P and [1]H magnetic resonance spectroscopy metabolomics techniques, which were consistent with our findings regarding the level of phosphoethanolamine (PE). However, the detailed mechanism might be related to the upregulation of diacylglycerol-acyltransferase 1 by glioma cells.^[36]^ The normal function of mitochondria in the brain is essential for the physiological role of neurons, and its alteration has been regarded as a critical hallmark of various cancer types. Kiebish et al observed that mice with metabolic abnormalities of phosphocholine (PC) and PE in the brain were more susceptible to spontaneous glioma.^[37]^

Recently, the sphingolipid system has been promoted as a targeting pathway for glioblastoma therapy.^[38]^ Various molecules of this pathway play roles ranging from activation to suppression in the pathogenesis of glioblastoma.^[39]^ In our study, phytosphingosine has been demonstrated to induce the autophagy of glioma.^[40]^ Ceramide and sphingomyelin can promote apoptosis of glioma,^[41,42]^ while galabiosylceramide has the potential to participate in killing glioma cells by dendritic and natural killer cells.^[43]^ In addition, derivatized sphingolipids have been reported to be at a high level in high-grade gliomas.^[44]^ To date, only the study by Sullards et al in 2003 systematically evaluated the detailed biological function of various sphingolipids in vitro.^[45]^ In the present study, perturbations of glycerophospholipid metabolism and systematic changes in sphingolipid metabolism were observed in patients with AA. The dysregulation of these pathways is highly consistent with the prediction model of metabolic profile.^[46]^ However, more detailed mechanisms of these differentiated compounds in the pathogenesis of AA require further experimental confirmation.

Linoleic acids, which are important polyunsaturated fatty acids, regulate the cell cycle, including peroxidation activities and apoptosis.^[47,48]^ In a previous study, the direct effects of linoleic acids on AA tumor cells varied and were specific to the tumor type,^[49]^ which is similar to the results of our study on the level of linoleic acids. The current study observed opposite trends in linoleic and bovinic acid in patients with AA. Gamma-Linolenic acid has been recently demonstrated to alter the migration, proliferation, and apoptosis of glioblastoma cells.^[50]^ The exhaustion of these bioactive molecules may be responsible for the deterioration of AA.^[51]^ A homolog of PC (16:0/16:0), which is involved in several metabolic pathways, including linoleic acid and glycerophospholipid, was also noticed to be markedly perturbed in AA, although the mechanism remains unknown.

Caffeine metabolism has been reported to participate in the regulation of signaling pathways associated with cellular apoptosis of glioma, including activation of cathepsin B, mitogen-activated protein kinase, and caspase-3.^[52,53]^ In addition, the metabolites of the caffeine metabolite pathway may regulate the activity, cellular cycle, and proliferation of glioma negatively.^[54]^ The present study identified for the first time the potential association between xanthine, 3-methylxanthine, theobromine, paraxanthine, and the pathogenesis of AA. These compounds could potentially prevent malignant glioblastoma proliferation by negatively regulating phosphodiesterase-4, extracellular signal-regulated kinase, Akt/mammalian target of rapamycin kinase, and nuclear factor kappa-light-chain-enhancer of activated B cells.^[55]^

Porphyrin metabolism perturbed with changes in several metabolites was also observed in AA in this study. Emerging evidence shows that protoporphyrin IX,^[56,57]^ as an intermediate compound of the heme-biosynthesis cascade, can produce fluorescence and is used as a standard for surgical resection of glioma,^[58]^ since it accumulates within the tumor tissues.^[59]^ Bilirubin has been demonstrated to activate endoplasmic reticulum stress-induced autophagy of neuronal cells, which is a critical method of apoptosis of tumor cells.^[60,61]^ The other distinct metabolites in the porphyrin metabolic pathway have rarely been reported to participate in the pathogenesis of AA or other gliomas. The potential regulatory and biological roles of these novel markers on the tumor cells of AA may provide new insights into the pathogenesis of AA.

Importantly, the present study identified several biomarkers for precision diagnosis. Of the 24 metabolites, 4, including 3-methylxanthine, sphinganine, LysoPC(18:1), and lactosylceramide, displayed an excellent predictive effect for AA. These biomarkers are bioactive molecules and are in critical topological positions of the metabolic network. However, the regulatory mechanism and detailed biological role of these biomarkers in the pathogenesis of AA needs further study. Tissue-specific energy metabolism dysfunction was not observed at the system level compared with the previous study.^[62]^ Given the small sample size of this study, which is the most important limitation of our study, a larger patient cohort needs to be included in future studies to test the accuracy of other metabolites for phenotyping. Due to the extremely limited quantitative ability of untargeted metabolomics techniques, a targeted metabolomics experiment is required for further investigation. In addition, genetic and pathogen background-controlled murine models could be fully utilized in the future to further explore the mechanism of AA. A quantitatively accurate, larger sample size-based targeted metabolomics study could also correlate with clinical information more confidently to mine the in-depth biological implications. In the present study, we performed a cross-sectional study on the mechanism of AA in samples from different patients. However, assessment of the metabolic profile of AA patients using a longitudinal method is very informative. It will be helpful to explore the mechanism of AA by determining the metabolic profile change in a longitudinal follow-up study in the future. Comparison of different cancers via metabolomics will also be another direction to elucidate the differentiated mechanism and unique features of various cancers.

## 5. Conclusion

The perturbed metabolic pattern related to immune regulation and cellular signaling transduction is associated with the pathogenesis of AA. 3-Methylxanthine, sphinganine, LysoPC(18:1), and lactosylceramide could be used as biomarkers of AA in future clinical practice. This study provides a therapeutic basis for further studies on the mechanism and clinical precision diagnosis of AA. Targeted metabolomics verification based on a larger sample cohort is necessary to improve the diagnosis strategy.

## Author contributions

CD participated in and validated the experiments, reanalyzed the data, finished the plotting, ZH performed the experiments, BW collected the samples and interpreted the data, and ML deigned and approved the current study. All authors have read and approved the final version of the manuscript.

## References

[1] Louis DN Ohgaki H Wiestler OD. The 2007 WHO classification of tumours of the central nervous system. Acta Neuropathol. 2007;114:97–109. 17618441 10.1007/s00401-007-0243-4 PMC1929165

[2] Grimm SA Chamberlain MC. Anaplastic astrocytoma. CNS Oncol. 2016;5:145–57. 27230974 10.2217/cns-2016-0002 PMC6042632

[3] Takenaka MC Gabriely G Rothhammer V. Control of tumor-associated macrophages and T cells in glioblastoma via AHR and CD39. Nat Neurosci. 2019;22:729–40. 30962630 10.1038/s41593-019-0370-y PMC8052632

[4] Quant EC Drappatz J Wen PY. Recurrent high-grade glioma. Curr Treat Options Neurol. 2010;12:321–33. 20842591 10.1007/s11940-010-0078-5

[5] Liu R Li W Tao B. Tyrosine phosphorylation activates 6-phosphogluconate dehydrogenase and promotes tumor growth and radiation resistance. Nat Commun. 2019;10:991. 30824700 10.1038/s41467-019-08921-8 PMC6397164

[6] Scobie MR Houke HR Rice CD. Modulation of glioma-inflammation crosstalk profiles in human glioblastoma cells by indirubin-3’-(2,3 dihydroxypropyl)-oximether (E804) and 7-bromoindirubin-3’-oxime (7BIO). Chem Biol Interact. 2019;312:108816. 31505164 10.1016/j.cbi.2019.108816

[7] Chang WH Lai AG. The pan-cancer mutational landscape of the PPAR pathway reveals universal patterns of dysregulated metabolism and interactions with tumor immunity and hypoxia. Ann N Y Acad Sci. 2019;1448:65–82. 31215667 10.1111/nyas.14170

[8] Crunkhorn S. Targeting cancer cell metabolism in glioblastoma. Nat Rev Cancer. 2019;19:250. 30944412 10.1038/s41568-019-0139-3

[9] Keskin DB Anandappa AJ Sun J. Neoantigen vaccine generates intratumoral T cell responses in phase Ib glioblastoma trial. Nature. 2019;565:234–9. 30568305 10.1038/s41586-018-0792-9 PMC6546179

[10] Cairncross JG Wang M Jenkins RB. Benefit from procarbazine, lomustine, and vincristine in oligodendroglial tumors is associated with mutation of IDH. J Clin Oncol. 2014;32:783–90. 24516018 10.1200/JCO.2013.49.3726 PMC3940537

[11] Pang Z Wang G Wang C. Serum metabolomics analysis of asthma in different inflammatory phenotypes: a cross-sectional study in Northeast China. Biomed Res Int. 2018;2018:2860521. 30345296 10.1155/2018/2860521 PMC6174811

[12] Banyou M Xiwu H Yadong L. MiR-124 inhibits malignant biological behaviors of glioma cells by targeting SDCBP. Int J Clin Exp Med. 2019;12:7.

[13] Pang Z Chong J Li S. MetaboAnalystR 3.0: toward an optimized workflow for global metabolomics. Metabolites. 2020;10:186. 10.3390/metabo10050186 PMC7281575 32392884

[14] White K Connor K Clerkin J. New hints towards a precision medicine strategy for IDH wild-type glioblastoma. Ann Oncol. 2020;31:1679–92. 32918998 10.1016/j.annonc.2020.08.2336

[15] Pandey R Caflisch L Lodi A. Metabolomic signature of brain cancer. Mol Carcinog. 2017;56:2355–71. 28618012 10.1002/mc.22694 PMC5708886

[16] Jothi J Janardhanam VA Krishnaswamy R. Metabolic variations between low-grade and high-grade gliomas—profiling by 1H NMR spectroscopy. J Proteome Res. 2020;19:2483–90. 32393032 10.1021/acs.jproteome.0c00243

[17] Pang Z Wang G Ran N. Inhibitory effect of methotrexate on rheumatoid arthritis inflammation and comprehensive metabolomics analysis using Ultra-Performance Liquid Chromatography-Quadrupole Time of Flight-Mass Spectrometry (UPLC-Q/TOF-MS). Int J Mol Sci . 2018;19. 10.3390/ijms19102894 PMC6212996 30249062

[18] Wishart DS Feunang YD Marcu A. HMDB 4.0: the human metabolome database for 2018. Nucleic Acids Res. 2018;46:D608–17. 29140435 10.1093/nar/gkx1089 PMC5753273

[19] Guijas C Montenegro-Burke JR Domingo-Almenara X. METLIN: a technology platform for identifying knowns and unknowns. Anal Chem. 2018;90:3156–64. 29381867 10.1021/acs.analchem.7b04424 PMC5933435

[20] Kanehisa M Furumichi M Tanabe M. KEGG: new perspectives on genomes, pathways, diseases and drugs. Nucleic Acids Res. 2017;45:D353–61. 27899662 10.1093/nar/gkw1092 PMC5210567

[21] Shannon P Markiel A Ozier O. Cytoscape: a software environment for integrated models of biomolecular interaction networks. Genome Res. 2003;13:2498–504. 14597658 10.1101/gr.1239303 PMC403769

[22] Aghajan Y Malicki DM Levy ML. Atypical anaplastic astrocytoma with unique molecular features and diffuse leptomeningeal spread in a child with long-term survival. BMJ Case Rep. 2019;12:e228153. 10.1136/bcr-2018-228153 PMC6381959 30765449

[23] Stegmann S Werner JM Kuhl S. Death receptor 6 (DR6) is overexpressed in astrocytomas. Anticancer Res. 2019;39:2299–306. 31092421 10.21873/anticanres.13346

[24] Luo W Yan D Song Z. miR-126-3p sensitizes glioblastoma cells to temozolomide by inactivating Wnt/beta-catenin signaling via targeting SOX2. Life Sci. 2019;226:98–106. 30980849 10.1016/j.lfs.2019.04.023

[25] Kesarwani P Prabhu A Kant S. Metabolic remodeling contributes towards an immune-suppressive phenotype in glioblastoma. Cancer Immunol Immunothe CII. 2019;68:1107–20. 10.1007/s00262-019-02347-3 PMC6586493 31119318

[26] Chinnaiyan P Kensicki E Bloom G. The metabolomic signature of malignant glioma reflects accelerated anabolic metabolism. Cancer Res. 2012;72:5878–88. 23026133 10.1158/0008-5472.CAN-12-1572-T PMC4638418

[27] Bjorkblom B Wibom C Jonsson P. Metabolomic screening of pre-diagnostic serum samples identifies association between alpha- and gamma-tocopherols and glioblastoma risk. Oncotarget. 2016;7:37043–53. 27175595 10.18632/oncotarget.9242 PMC5095057

[28] Cuperlovic-Culf M Ferguson D Culf A. 1H NMR metabolomics analysis of glioblastoma subtypes: correlation between metabolomics and gene expression characteristics. J Biol Chem. 2012;287:20164–75. 22528487 10.1074/jbc.M111.337196 PMC3370199

[29] Reitman ZJ Jin G Karoly ED. Profiling the effects of isocitrate dehydrogenase 1 and 2 mutations on the cellular metabolome. Proc Natl Acad Sci USA. 2011;108:3270–5. 21289278 10.1073/pnas.1019393108 PMC3044380

[30] Xu W Yang H Liu Y. Oncometabolite 2-hydroxyglutarate is a competitive inhibitor of alpha-ketoglutarate-dependent dioxygenases. Cancer Cell. 2011;19:17–30. 21251613 10.1016/j.ccr.2010.12.014 PMC3229304

[31] Christians A Adel-Horowski A Banan R. The prognostic role of IDH mutations in homogeneously treated patients with anaplastic astrocytomas and glioblastomas. Acta Neuropathol Commun. 2019;7:156. 31623667 10.1186/s40478-019-0817-0 PMC6798425

[32] Wick W Meisner C Hentschel B. Prognostic or predictive value of MGMT promoter methylation in gliomas depends on IDH1 mutation. Neurology. 2013;81:1515–22. 24068788 10.1212/WNL.0b013e3182a95680

[33] Bonacina F Coe D Wang G. Myeloid apolipoprotein E controls dendritic cell antigen presentation and T cell activation. Nat Commun. 2018;9:3083. 30082772 10.1038/s41467-018-05322-1 PMC6079066

[34] Barthomeuf C Lamy S Blanchette M. Inhibition of sphingosine-1-phosphate- and vascular endothelial growth factor-induced endothelial cell chemotaxis by red grape skin polyphenols correlates with a decrease in early platelet-activating factor synthesis. Free Radic Biol Med. 2006;40:581–90. 16458188 10.1016/j.freeradbiomed.2005.09.015

[35] Willems E Dedobbeleer M Digregorio M. Aurora A plays a dual role in migration and survival of human glioblastoma cells according to the CXCL12 concentration. Oncogene. 2019;38:73–87. 30082913 10.1038/s41388-018-0437-3 PMC6755987

[36] Cheng X Geng F Pan M. Targeting DGAT1 ameliorates glioblastoma by increasing fat catabolism and oxidative stress. Cell Metab. 2020;32:229–242.e8. 32559414 10.1016/j.cmet.2020.06.002 PMC7415721

[37] Kiebish MA Han X Cheng H. Brain mitochondrial lipid abnormalities in mice susceptible to spontaneous gliomas. Lipids. 2008;43:951–9. 18560917 10.1007/s11745-008-3197-y PMC3773477

[38] Tea MN Poonnoose SI Pitson SM. Targeting the sphingolipid system as a therapeutic direction for glioblastoma. Cancers. 2020;12:111. 10.3390/cancers12010111 PMC7017054 31906280

[39] Bottai D Adami R Paroni R. Brain cancer-activated microglia: a potential role for sphingolipids. Curr Med Chem. 2020;27:4039–61. 31057101 10.2174/0929867326666190506120213

[40] Casasampere M Ordonez YF Casas J. Dihydroceramide desaturase inhibitors induce autophagy via dihydroceramide-dependent and independent mechanisms. Biochim Biophys Acta Gen Subj. 2017;1861:264–75. 27894925 10.1016/j.bbagen.2016.11.033

[41] Sordillo LA Sordillo PP Helson L. Sphingosine kinase inhibitors as maintenance therapy of glioblastoma after ceramide-induced response. Anticancer Res. 2016;36:2085–95. 27127108

[42] Magrassi L Adorni L Montorfano G. Vitamin D metabolites activate the sphingomyelin pathway and induce death of glioblastoma cells. Acta Neurochir (Wien). 1998;140:707–13; discussion 713 9781285 10.1007/s007010050166

[43] Liu H Chen L Liu J. Co-delivery of tumor-derived exosomes with alpha-galactosylceramide on dendritic cell-based immunotherapy for glioblastoma. Cancer Lett. 2017;411:182–90. 28947140 10.1016/j.canlet.2017.09.022

[44] Woo SR Oh YT An JY. Glioblastoma specific antigens, GD2 and CD90, are not involved in cancer stemness. Anat Cell Biol. 2015;48:44–53. 25806121 10.5115/acb.2015.48.1.44 PMC4371180

[45] Sullards MC Wang E Peng Q. Metabolomic profiling of sphingolipids in human glioma cell lines by liquid chromatography tandem mass spectrometry. Cell Mol Biol (Noisy-le-grand). 2003;49:789–97. 14528916

[46] Righi V Cavallini N Valentini A. A metabolomic data fusion approach to support gliomas grading. NMR Biomed. 2019. 10.1002/nbm.4234 31825557

[47] Nasrollahzadeh J Siassi F Doosti M. The influence of feeding linoleic, gamma-linolenic and docosahexaenoic acid rich oils on rat brain tumor fatty acids composition and fatty acid binding protein 7 mRNA expression. Lipids Health Dis. 2008;7:45. 19014610 10.1186/1476-511X-7-45 PMC2605445

[48] Viita H Pacholska A Ahmad F. 15-Lipoxygenase-1 induces lipid peroxidation and apoptosis, and improves survival in rat malignant glioma. In Vivo. 2012;26:1–8. 22210710

[49] Maggiora M Bologna M Ceru MP. An overview of the effect of linoleic and conjugated-linoleic acids on the growth of several human tumor cell lines. Int J Cancer. 2004;112:909–19. 15316938 10.1002/ijc.20519

[50] Andreoli Miyake J Nascimento Gomes R Colquhoun A. Gamma-Linolenic acid alters migration, proliferation and apoptosis in human and rat glioblastoma cells. Prostagland Other Lipid Mediators. 2020;150:106452. 10.1016/j.prostaglandins.2020.106452 32439412

[51] Das UN. Molecular Mechanism of Anti-cancer Action of PUFAs with Particular Reference to GLA in Glioma: Springer US, 2020.

[52] Cheng YC Ding YM Hueng DY. Caffeine suppresses the progression of human glioblastoma via cathepsin B and MAPK signaling pathway. J Nutr Biochem. 2016;33:63–72. 27260469 10.1016/j.jnutbio.2016.03.004

[53] Liu JD Song LJ Yan DJ. Caffeine inhibits the growth of glioblastomas through activating the caspase-3 signaling pathway in vitro. Eur Rev Med Pharmacol Sci. 2015;19:3080–8. 26367732

[54] Jiang J Lan YQ Zhang T. The in vitro effects of caffeine on viability, cycle profiles, proliferation, and apoptosis of glioblastomas. Eur Rev Med Pharmacol Sci. 2015;19:3201–7. 26400523

[55] Sugimoto N Miwa S Hitomi Y. Theobromine, the primary methylxanthine found in Theobroma cacao, prevents malignant glioblastoma proliferation by negatively regulating phosphodiesterase-4, extracellular signal-regulated kinase, Akt/mammalian target of rapamycin kinase, and nuclear factor-kappa B. Nutr Cancer. 2014;66:419–23. 24547961 10.1080/01635581.2013.877497

[56] Labuschagne J. 5-aminolevulinic acid-guided surgery for focal pediatric brainstem gliomas: a preliminary study. Surg Neurol Int. 2020;11:334–334. 33194268 10.25259/SNI_246_2020 PMC7656004

[57] Charalampaki P Proskynitopoulos PJ Heimann A Nakamura M. 5-Aminolevulinic acid multispectral imaging for the fluorescence-guided resection of brain tumors: a prospective observational study. Front Oncol. 2020;10. 10.3389/fonc.2020.01069 PMC7362891 32733798

[58] Maugeri R Villa A Pino M. With a little help from my friends: the role of intraoperative fluorescent dyes in the surgical management of high-grade gliomas. Brain Sci. 2018;8. 10.3390/brainsci8020031 PMC5836050 29414911

[59] Kroger S Niehoff AC Jeibmann A. Complementary molecular and elemental mass-spectrometric imaging of human brain tumors resected by fluorescence-guided surgery. Anal Chem. 2018;90:12253–60. 30215510 10.1021/acs.analchem.8b03516

[60] Qaisiya M Mardesic P Pastore B. The activation of autophagy protects neurons and astrocytes against bilirubin-induced cytotoxicity. Neurosci Lett. 2017;661:96–103. 28965934 10.1016/j.neulet.2017.09.056

[61] Qaisiya M Brischetto C Jasprova J. Bilirubin-induced ER stress contributes to the inflammatory response and apoptosis in neuronal cells. Arch Toxicol. 2017;91:1847–58. 27578021 10.1007/s00204-016-1835-3

[62] Levenson CW Morgan TJ Twigg PD. Use of MRI, metabolomic, and genomic biomarkers to identify mechanisms of chemoresistance in glioma. Cancer Drug Resist. 2019;2:862–76. 35582585 10.20517/cdr.2019.18 PMC8992521

